# Correction: The detection of oral pre- malignant lesions with an autofluorescence based imaging system (VELscope^TM^) – a single blinded clinical evaluation

**DOI:** 10.1186/1746-160X-9-26

**Published:** 2013-09-10

**Authors:** Henning Hanken, Juliane Kraatz, Ralf Smeets, Max Heiland, Alexandre Thomas Assaf, Marco Blessmann, Wolfgang Eichhorn, Till Sebastian Clauditz, Alexander Gröbe, Andreas Kolk, Madiha Rana

**Affiliations:** 1 Department of Oral and Maxillofacial Surgery, University Medical Center Hamburg-Eppendorf, Martinistraße 52, 20246, Hamburg, Germany; 2 Institute of Pathology, University Medical Center Hamburg-Eppendorf, Hamburg, Germany; 3 Department of Oral and Maxillofacial Surgery, Technische Universität München, Klinikum rechts der Isar, Munich, Germany; 4 Department of Differential Psychology and Psychological Assessment, Helmut-Schmidt-University, University of the Federal Armed Forces, Hamburg, Germany

## Correction

After publication of this work [1], we noted that we inadvertently failed to include the complete list of all coauthors. The full list of authors has now been added and the Authors’ contributions, Competing interests and Acknowledgment section have been modified accordingly.

## Competing interests

The authors declare that they have no competing interests.

## Authors’ contributions

HH, JK, RS, MH, AA, MB, WE, TC, AG, AK and MR conceived of the study and participated in its design and coordination. JK and HH made substantial contributions to conception and design of the manuscript as well as data acquisition. MH, MB, TC, AG have been involved in drafting the manuscript. RS and MR were involved in revising the manuscript. All authors read and approved the final manuscript.
